# The evolution of the Banff classification schema for diagnosing renal allograft rejection and its implications for clinicians

**DOI:** 10.4103/0971-4065.62086

**Published:** 2010

**Authors:** D. M. Bhowmik, A. K. Dinda, P. Mahanta, S. K. Agarwal

**Affiliations:** Department of Nephrology, All India Institute of Medical Sciences, New Delhi, India; 1 Department of Pathology, All India Institute of Medical Sciences, New Delhi, India

**Keywords:** Banff classification, renal allograft biopsy, updates

## Abstract

Till the early 1990s there was no standardized international classification of renal allograft biopsies resulting in considerable heterogeneity in reporting among the various centers. A group of dedicated renal pathologists, nephrologists, and transplant surgeons developed a schema in Banff, Canada in 1991. Subsequently there have been updates at regular intervals. The following review presents the evolution of the Banff classification and its utility for clinicians.

## Introduction

Until the early 1990s, rejection of the renal allograft was classically classified into the following four types: 1) hyperacute, 2) acute, 3) accelerated acute, and 4) chronic.[1] Hyperacute rejection was defined as immediate (minutes to hours) rejection of the graft due to the presence of preformed antibodies. Acute rejection was defined as rejection occurring after 5–7 days due to activated T cells. Accelerated acute was defined as an aggressive episode of rejection occurring within five days, due to formation of antibodies in a presensitized patient (history of blood transfusion, pregnancy, earlier transplant). Chronic rejection was defined as slow progressive graft dysfunction starting after three months. The pathophysiology of chronic rejection was considered to be both immune and nonimmune mediated. However, there was considerable heterogeneity among pathologists in characterization of renal allograft biopsies. Hence, it was felt that standardization of renal allograft biopsy was necessary to guide therapy and to help establish an objective end-point in clinical trials similar to that in heart and lung transplantation. Hence, a group of pathologists, nephrologists, and transplant surgeons met in Banff Canada from 2–4 August, 1991, to formulate a schema for nomenclature and classification of renal allograft pathology. The schema underwent considerable revision over the next couple years through follow-up meetings and correspondence including circulation of slides; and finally published in March 1993.[2] Thereafter, the Banff group has been meeting at regular intervals. So far there have been a total of 10 Banff conferences, with publication of four updates of the original classification, the latest being in April 2008.[3–6] The Banff schema has undergone considerable evolution over the last 15 years. Besides Banff, other classification schemes have also been studied.[7,8] All these are somewhat confusing for the nephrologist. Hence, an attempt has been made to present the evolution of the various classification systems over the last 15 years in a form useful to nephrologists [Table 1].

**Table 1:** Evolution of the histopathological classification of renal allograft rejection

Pre-Banff[1]	1^st^ Banff[2]	Banff'97[3]	Banff'97 Update[4]	Banff'051[5]	Banff'07[6]
1. Normal	1. Normal	1. Normal	1. Normal	1. Normal	1. Normal
2. Hyperacute	2. Hyperacute	2. Antibody-mediated rejection immediate – Hyperacute Delayed – accelerated acute	2. Antibody-mediated rejection	2. Antibody-mediated rejection	2. Antibody-mediated rejection
			Type I: C4d+, ATN, min. inflamm Type II: C4d+, leukocytes in ptc Type III: C4d+, transmural arteritis	Acute AMR Type I: C4d+, ATN, min. inflamm Type II: C4d+, leukocytes in ptc Type III: C4d+, Transmural arteritis Chronic active AMR	Acute AMR Type I: C4d+, ATN, min. inflamm Type II: C4d+, leukocytes in ptc Type III: C4d+, transmural arteritis Chronic active AMR
3. Accelerated acute	3. Borderline Mild tubulitis: t0, t1 interstitial inflamm: i0, i1	3. Borderline Mild tubulitis: t0, t1 interstitial inflamm: i0, i1	3. Borderline Mild tubulitis: t0, t1 interstitial inflamm: i0, i1	3. Borderline Mild tubulitis: t0, t1 interstitial inflamm: i0, i1	3. Borderline Mild tubulitis: t0, t1 interstitial inflamm: i0, i1
4. Acute rejection	4. Acute rejection Grade I: i2–i3 and/ or t2 Grade II: t3 and/or intimal arteritis: v1, v2 Grade III: transmural arteritis v3	4. Acute/Active rejection Type IA: i2, i3 & t2 Type IB: severe tubulitis t3 Type IIA: mild-mod intimal arteritis v1 Type IIB: severe intimal arteritis v2 Type III: transmural arteritis v3	4. Acute/Active cellular rejection Type IA: i2, i3 & t2 Type IB: severe tubulitis t3 Type IIA: mild-mod intimal arteritis v1 Type IIB: severe intimal arteritis v2 Type III: transmural arteritis v3	4. T-cell-mediated rejection Acute TCR Type IA: i2, i3 & t2 Type IB: severe tubulitis t3 Type IIA: mild- mod intimal arteritis v1 Type IIB: severe intimal arteritis v2 Type III: transmural arteritis v3 Chronic active TCR	4. T-cell-mediated rejection Acute TCR Type IA: i2, i3 & t2 Type IB: severe tubulitis t3 Type IIA: mild-mod intimal arteritis v1 Type IIB: severe intimal arteritis v2 Type III: transmural arteritis v3 Chronic active TCR
5. Chronic rejection	5. Chronic allograft nephropathy Grade I: mild Grade II:moderate Grade III: severe	5. Chronic allograft nephropathy Grade I: mild Grade II:moderate Grade III: severe	5. Chronic allograft nephropathy Grade I: mild Grade II:moderate Grade III: severe	5. Interstitial fibrosis and tubular atrophy (IFTA) Grade I: mild Grade II:moderate Grade III: severe	5. Interstitial fibrosis and tubular atrophy (IFTA) Grade I: mild Grade II:moderate Grade III: severe
	6. Other: Changes not due to rejection	6. Other: Changes not due to rejection	6. Other: Changes not due to rejection	6. Other: Changes not due to rejection	6. Other: Changes not due to rejection

Mononuclear cell interstitial inflammation ("i") score; i0-No or trivial inflammation (<10% of unscarred parenchyma); i1-10–25% of parenchyma inflamed; i2-26–50% of parenchyma inflamed; i3-more than 50% of parenchyma inflamed; Tubulitis ("t") score; t0: No mononuclear cells in tubules; t1-1–4 cells/tubular cross section; t2-5–10 cells/tubular cross section; t3 > 10 cells/tubular cross section; or tubular basement membrane destruction with i2/i3 inflammation Intimal arteritis ("v") score;v0-No arteritis; v1-Mild to moderate intimal arteritis; v2-Severe intimal arteritis; v3-Transmural arterits and/or fibrinoid necrosis

## First Banff

As mentioned earlier, the aim of first Banff schema published in 1993[2] was to standardize the histopathological diagnosis of renal allograft biopsy. Specimen adequacy was taken as > 7 glomeruli with at least one artery. It was recommended to have seven slides:3 H and E, 3 PAS, and 1 trichrome. The novel features comprised introduction of the categories ‘Borderline Changes’ and ‘Chronic Allograft Nephropathy’ (CAN); and *grading* ‘acute rejection’ into mild, moderate, and severe. Banff also introduced a numerical grading system for each of the renal compartments-interstitium (i), tubules (t), vessels (v), and glomeruli (g) as: 0-absent, 1-mild, 2-moderate, and 3-severe. On histopathology, the renal allograft biopsies were classified into six categories *viz*., normal, hyperacute rejection, borderline, acute rejection, chronic allograft nephropathy, and other i.e. changes not due to rejection.

### Hyperacute rejection

Hyperacute rejection occurs within 10 minutes to 1 hour after perfusion with the recipient's blood. This occurs since the recipient is presensitized to alloantigens on the surface of the graft endothelium.[9] These alloantigens include:

- ABO incompatibility: Primarily IgM antibodies.
- Anti-HLA class I antibodies: Primarily IgG3.
- Anti-HLA class II antibodies: IgG/IgM antibodies in glomeruli and peritubular capillaries (where class II is prominent).
- Anti-endothelial-monocyte antibodies.

During surgery, the initial pink kidney becomes soft, flabby, mottled purple or cyanotic and anuric. Subsequently, it swells with widespread interstitial hemorrhage and cortical necrosis. The histological features include the presence of thrombi in the microvasculature, interstitial hemorrhages, and prominence of neutrophils in the glomeruli. Currently, it is well known that C4d staining in peritubular capillaries is the diagnostic feature, which helps in differentiating it from vascular thrombosis. Hyperacute rejection is now rare, seen in nearly 0.5% of transplants. In addition, some cases of primary nonfunction of the graft may be due to hyperacute rejection. As it is well known that the treatment of hyperacute rejection is nephrectomy.

### Borderline changes

Borderline changes was characterized by infiltration of mononuclear cells (<25% of the parenchyma) or foci of mild tubulitis (1–4 mononuclear cells/tubular cross-section). Borderline changes might be considered suggestive of ‘very mild acute rejection’, but which were nondiagnostic. Banff opined that it was not mandatory to treat Borderline rejection. This has been somewhat controversial.

### Acute rejection

It was felt that tubulitis was a better measure of severity of rejection than the intensity or extent of interstitial lymphocytic infiltration. Thus, tubulitis (infiltration by > 4 mononuclear cells / tubular cross-section) and intimal arteritis (subendothelial infiltration by mononuclear cells) were taken as defining features for acute rejection. Glomerulitis, although included in the scoring system, was not taken for diagnosing or grading of acute rejection. Acute rejection was graded as follows:

Grade I acute rejection: Moderate (>25%) to severe mononuclear cell interstitial infiltrate (i2/i3) and moderate tubulitis (4 mononuclear cells/tubular cross section i.e., t2).

Grade II acute rejection: Severe tubulitis (t3) and/or intimal arteritis (v1/v2).

Grade III acute rejection: Transmural arteritis (v3).

### Chronic allograft nephropathy

Banff introduced the category ‘chronic allograft nephropathy’ (CAN) as a histopathological correlate of chronic allograft dysfunction. CAN was thought to include at least four entities at that period of time *viz*. chronic rejection, chronic cyclosporine toxicity, hypertensive changes, and chronic infection. The features suggestive of chronic rejection were: a) chronic transplant glomerulopathy: Glomerular basement membrane duplication and mesangial cell proliferation, and b) vasculopathy: Fibrous intimal thickening often with fragmentation of internal elastic lamina. Chronic changes in the interstitium (ci), tubules (ct), vessels (cv), and glomerulus (cg) were likewise graded into 0, 1, 2, and 3. The severity of interstitial fibrosis and tubular atrophy, as also chronic transplant glomerulopathy and vasculopathy were used to grade CAN into mild, moderate, and severe.Two other classification systems which developed around this time deserve mention.

### Chronic allograft damage index

Chronic allograft damage index (CADI) score was first developed in 1994.[7] The CADI score was obtained by scoring each of the following from 0–3: Diffuse or focal inflammation, interstitial fibrosis, increase in mesangial matrix, glomeruloscerosis, intimal proliferation of vessels and tubular atrophy. CADI score < 2 is associated with a good graft survival, while high CADI score > 4 is associated with a poor outcome.[10] This scoring system is still used occasionally both to study protocol and indication biopsies.

### Cooperative clinical trials in transplantation system

In 1997, pathologists in centers participating in the cooperative clinical trials in transplantation (CCTT) sponsored by the National Institutes of Health developed a scoring system (based on Banff) for the diagnosis of acute rejection. This was named as the CCTT system,[8] which was more simple and objective. It replaced ‘grades of rejection’ in the Banff schema by ‘types of rejection’. There was no category of ‘borderline rejection’; and all cases of borderline rejection as per Banff met the criteria for type I rejection in the CCTT system. Type I rejection was defined by mononuclear interstitial infiltrate (>5% of parenchyma) and tubulitis. It recognized the importance of vasculitis per sec, as it has implications for response to therapy and graft survival. Type II rejection was defined by arterial or arteriolar endothelialitis and type III by fibrinoid necrosis or transmural inflammation. These criteria were subsequently incorporated in the Banff '97 schema. Currently, the CCTT system is no longer used independently.

## Banff '97

**Figure 1:**
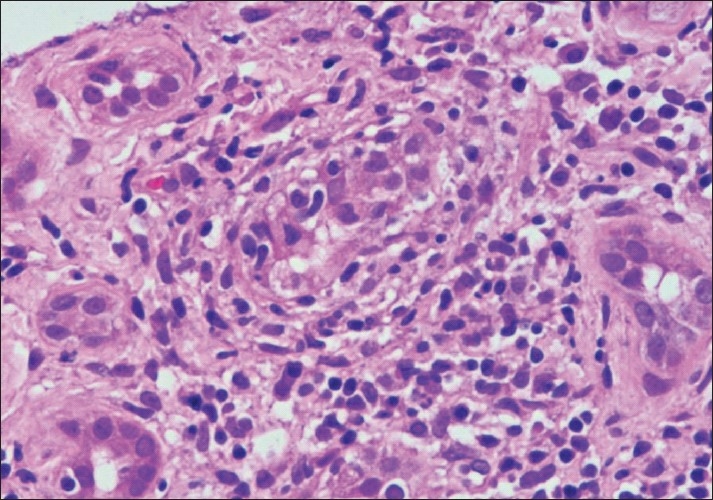
Photomicrograph showing a focus of tubulitis (t3) (H and E stain×200)

**Figure 2:**
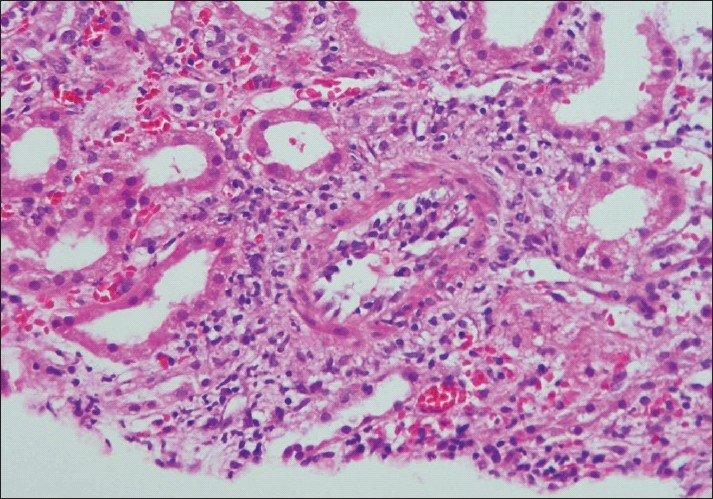
Photomicrograph of a small artery showing subintimal accumulation of mononuclear cells with about 50% narrowing of the luminal vascular area (Intimal arteritis v2) (H and E stain×100)

Banff '97 constitutes a landmark document in the field of renal transplant pathology.[3] It was developed using the First Banff schema and the CCTT modifications. The important changes introduced include:

- Adequacy of specimen: Two cores of tissue with cortex containing ≥10 glomeruli and at least twp arteries (bigger than arterioles), and section thickness 3–4 µm.
- Category 2: (Hyperacute rejection in the first Banff) was renamed as antibody-mediated rejection (AMR), which included two subcategories: A) hyperacute rejection, and B) accelerated acute rejection. The diagnosis of acute accelerated rejection was based on positive repeat cross match. It was also recognized that AMR may be super-imposed on acute cellular rejection.
- Category 3: Acute/active rejection: Many features of the CCTT classification were incorporated here. The grades of acute rejection were changed to type/grade. Recognizing the importance of vasculitis, both in response to treatment and long-term graft survival, the presence of intimal arteritis was taken as Type II acute rejection, while severe tubulitis without arteritis was reclassified as Type IB acute rejection. The classification of acute rejection is as under:
- Type (Grade) I: Tubulo-interstitial inflammation only. IA: Interstitial inflammation moderate-severe (i2,i3) and/or tubulitis moderate (t2). IB: Tubulitis severe (t3) [Figure 1].
- Type (Grade) II: Intimal arteritis. IIA: Intimal arteritis mild-moderate (v1). IIB: Intimal arteritis severe (v2) [Figure 2].
- Type (Grade) III: Transmural arteritis and/or fibrinoid necrosis (v3).

As mentioned later, this classification of acute rejection still holds good, although it is now placed under acute T-cell-mediated rejection.

## Banff '97 Update

In the 2001 Banff meeting, published in 2003, it was recognized that antibodies play an important role in causing rejection.[4] Studies presented at this Banff Conference showed that biopsies with C4d deposition had distinctly lower graft survival. Tubular HLA-DR expression was the morphologic feature most closely linked to deposition of C4d. Hence, C4d staining of the peritubular capillaries (ptc) was accepted as the marker of antibody-mediated rejection (AMR). Detailed pathological classification of AMR was outlined as follows:

- Type (Grade) I: C4d+, ATN like with minimal inflammation.
- Type (Grade) II: C4d+, capillary margination and/or thrombosis.
- Type (Grade) III: C4d+, transmural arteritis (v3).

The prior category of acute rejection was renamed as acute/active cellular rejection. Rest of the Banff '97 schema was left unchanged. More powerful immunosuppression was considered necessary in patients with AMR. Workers reported that use of plasmapheresis and IVIg was associated with good outcome. Use of anti-CD 20 therapy was also reported.

## Banff '05

The 8^th^ Banff Conference was held in July 2005 and published in 2007.[5] The major revisions were: 1) change in the basic classification of rejection from acute and chronic to its pathophysiologic basis *viz*. antibody- mediated (AMR) and T-cell-mediated (TCR), either of which could be acute or chronic; and 2) Elimination of CAN, which had actually been introduced by the First Banff 12 years back.

### Reasons for eliminating the terminology ‘CAN’

CAN is not a homogenous entity. It is the end result of various injurious processes to the glomerulus, tubulointerstitium, and the microvascular compartments; resulting in interstitial fibrosis and tubular atrophy. The various such causes, which may be present either singly or in combination are shown in [Table 2].[11] During the last 12 years since the introduction of the term, researchers used ‘CAN’ indiscriminately as a diagnostic entity by itself rather than making attempts to pinpoint the exact disease causing CAN. Hence, in 2003, the Banff consensus conference decided to eliminate the term CAN from the Banff schema. Instead all efforts should be made to diagnose the etiology of the chronic allograft injury. If attempts to find the exact cause fail, then the biopsy report should be labelled as ‘interstitial fibrosis and tubular atrophy-not otherwise specified’ as discussed below.

**Table 2:** Conditions causing chronic allograft dysfunction, which can be ascertained[11]

Chronic active antibody-mediated rejection
Chronic cell-mediated interstitial rejection
Calcineurin inhibitor toxicity
Hypertensive damage
BK virus nephropathy
Bacterial infections
Recurrent disease

The re-classified categories are as follows:

- Category 2: Antibody mediated rejection: This category includes acute and chronic active antibody mediated rejection
- Acute antibody-mediated rejection: Acute antibody-mediated rejection (also called acute humoral rejection) occurs when acute rejection occurs due to antidonor antibodies. AMR may occur on the operating table, or even weeks to months later. The histological features include ATN, presence of neutrophils in the peritubular capillaries, thrombi and fibrinoid necrosis, along with C4d deposition.[12] The grading of acute AMR remains the same as mentioned earlier. The term-hyperacute rejection-is not mentioned in the Banff schema since 2003, but still described in the standard textbooks.[9] However, the term accelerated acute rejection is confusing and should no longer be used.

AMR may be super-imposed on acute T-cell-mediated rejection. AMR needs stronger immunosuppression. Plasmapheresis, IVIg, and ant-CD 20 antibody rituximab have all been tried with variable success.

- Chronic active antibody-mediated rejection: This is also called chronic humoral rejection. In biopsies with interstitial fibrosis and tubular atrophy, the presence of additional glomerular and vascular features is suggestive of immune-mediated chronic rejection. The association between antidonor HLA antibodies and the development of chronic allograft dysfunction and obliterative vasculopathy was actually first demonstrated way back in 1970.[13] In the subsequent years, this idea remained in hibernation. It was three decades later that C4d deposition in peritubular capillaries was strongly associated with transplant glomerulopathy.[14] The features of chronic active antibody mediated rejection are shown in [Table 3]. On light microscopy, the important histopathological features are glomerular basement membrane duplication, multilamination of the peritubular capillary basement membrane and arterial intimal fibrosis. Two other conditions in which duplication of the glomerular basement membrane may occur are thrombotic microangiopathy and membranous glomerulopathy. It has been postulated that denovo membranous glomerulopathy may be related to chronic antibody-mediated rejection. The postulated stages of CHR are:[15]
- Development of donor specific antibodies
- Deposition of C4d in the allograft
- Graft pathology (subclinical rejection)
- Chronic graft dysfunction

**Table 3:** Criteria for diagnosis of chronic antibody mediated rejection[15]

Histopathology
Interstitial fibrosis and tubular atrophy associated with:
Transplant glomerulopathy
Glomerular basement membrane duplication
Increased mesangial matrix
Increased amount of endothelial cytoplasm
Loss of fenestrations
Transplant capillariopathy
Loss of peritubular capillaries resulting in reduced capillary density
Multilamination of peritubular capillary basement membrane
Transplant arteriopathy
Arterial intimal fibrosis with intimal monocyte cell infiltration.
Immunopathology
C4d deposition in peritubular capillaries and/or glomeruli
Serology
Anti-donor HLA or other endothelial antigens

Most cases of CHR are of insidious onset without past history of acute humoral rejection. CHR is present in about 10% of indication biopsies.

- Category 4: T-cell-Mediated Rejection: This category includes Acute and Chronic active T-cell-mediated rejection
  - Acute T-cell-mediated rejection: Acute T-cell mediated rejection occurs after 5–6 days. This time is required for the antigen presenting cells to present the alloantigens in the spleen and lymph nodes. There is interstitial infiltrate of mononuclear cells with interstitial edema and very occasionally hemorrhage. The infiltrate consists of mainly *t*-lymphocytes and macrophages. The presence of lymphoblasts (activated T cells) indicate increased proliferative activity.[9] The mononuclear cells invade tubules and can be seen between the tubular epithelial cells. This is termed as tubulitis and is best seen in slides stained with the PAS stain. *t*- cells express cytotoxic molecules viz. perforin, Fas ligand, granzyme A, and TNFβ. The classification of acute TCR is the same as ‘acute rejection’ mentioned in Banff '97 schema. Endarteritis (type II acute rejection) is manifested by the infiltration of mononuclear cells under the vascular endothelium. It affects arteries and arterioles. While under normal conditions the arterial endothelial cells express HLA class I antigens, during acute rejection they express increased HLA DR, ICAM-I and VCAM-I.[9] Glomerular lesions are uncommon in acute rejection. Very occasionally, ‘transplant glomerulitis’ may be seen. The endothelial cells are enlarged and hypercellular, with infiltration by mononuclear cells.

### Clinical significance of other types of cells in the interstitium

Although occasional eosinophils may be present, the presence of abundant eosinophils is associated with higher grades of rejection and a worse prognosis. The differential diagnosis consists of drug induced acute interstitial nephritis and infection. Plasma cells are associated with a poor graft survival.[16] Similarly the presence of CD 20 + B cells in the interstitium is associated with a poor response to steroids.[17] Neutrophils are suggestive of AMR or pyelonephritis. Mast cells correlate with interstitial edema.

**Figure 3:**
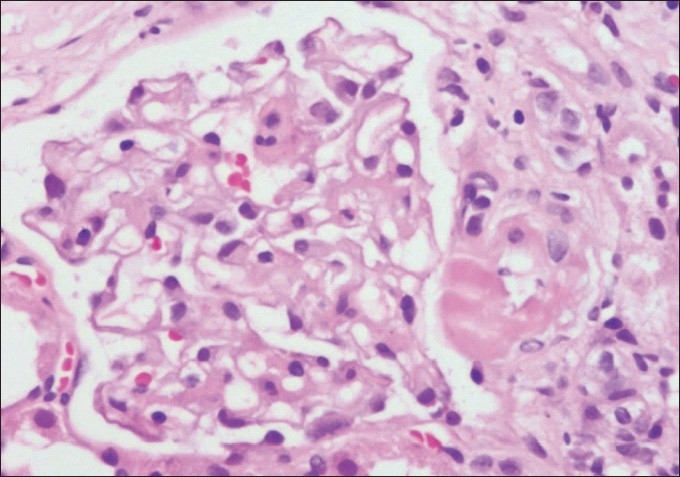
Photomicrograph showing a glomerulus with its afferent arteriole demonstrating a transmural hyaline nodule suggestive of CNI toxicity (H and E stain×200)

**Figure 4:**
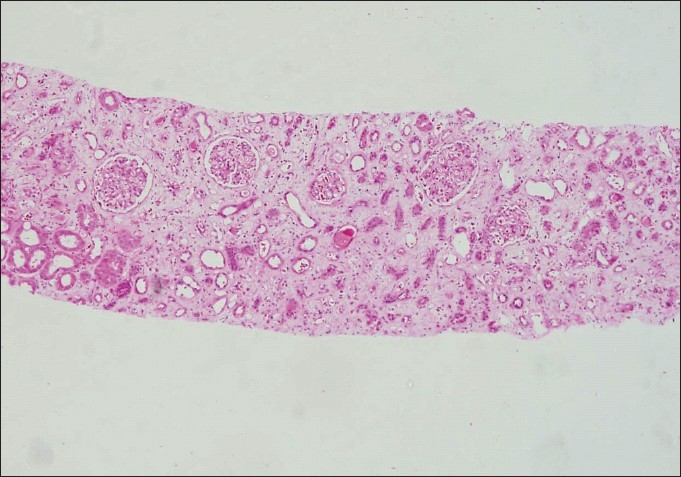
Low-power photomicrograph showing extensive tubular atrophy and interstitial fibrosis (IFTA Grade III) (H and E stain×40)

- Chronic active T-cell-mediated rejection: The defining feature is ‘chronic allograft arteriopathy’ characterized by arterial intimal fibrosis with mononuclear cell infiltration, and formation of neo-intima.
- Category 5: Interstitial fibrosis and tubular atrophy: IFTA replaces the term CAN in category 5. The diagnosis of IFTA should only be given if attempts to find the exact cause eg. antibody mediated, cell mediated, CNI toxicity [Figure 3], etc., of chronic histopathological changes in the allograft fail. The grading of IFTA is mild, moderate and severe [Figure 4].

## Banff '07

The significant updates include the following:

- Every renal allograft biopsy should be stained for C4d. C4d staining of the peritubular capillaries was graded: C4d0-negative, C4d1- minimal 1–10%, C4d2 – focal 10–50%, and C4d3 -diffuse > 50%.
- Peritubular capillaritis was graded.

The various categories remain unchanged.

### Impact of Banff classification

Mueller *et al*. confirmed a significant association between the revised Banff '97 classification and graft outcome.[18] Intimal arteritis was the only significant predictor of a poor survival probability. An area of some controversy is the ‘borderline change’. Although Banff recommends that treatment may not be indicated, studies have shown beneficial effect of treating this group with ant-rejection therapy.[18,19] Recently, SWOT (strengths, weaknesses, opportunities and threats) analysis of Banff was done. They found that Banff had universal impact on clinical practice and research.[20] Currently, for routine clinical purposes, pathologists mention only the broad Banff category. Detailed scoring of the various compartments of the renal parenchyma is not mentioned in the report.

### Future perspectives

During rejection certain genes-g interferon inducible or cytotoxic T-cell associated are upregulated.[21,22] A study of the transcriptional profile of these genes is called transcriptomics, which can improve the biopsy diagnosis. One area in which it is especially useful is-the Banff ‘Borderline rejection’. These cases can be resolved into two distinct classes *viz*., rejection and nonrejection.[22] It is expected that future Banff updates will integrate the histopathological and molecular parameters.
